# Mitochondria: Potential Targets for Osteoarthritis

**DOI:** 10.3389/fmed.2020.581402

**Published:** 2020-11-26

**Authors:** Xingjia Mao, Panfeng Fu, Linlin Wang, Chuan Xiang

**Affiliations:** ^1^Department of Orthopedic, The Second Hospital of Shanxi Medical University, Taiyuan, China; ^2^Department of Respiratory and Critical Care, The Affiliated Hospital of Medical School of Ningbo University, Ningbo, China; ^3^Department of Basic Medicine Sciences, The School of Medicine of Zhejiang University, Hangzhou, China

**Keywords:** osteoarthritis, mitochondria, mitochondrial dynamics mtDNA, biomedical therapy, bibliometrics

## Abstract

Osteoarthritis (OA) is a common and disabling joint disorder that is mainly characterized by cartilage degeneration and narrow joint spaces. The role of mitochondrial dysfunction in promoting the development of OA has gained much attention. Targeting endogenous molecules to improve mitochondrial function is a potential treatment for OA. Moreover, research on exogenous drugs to improve mitochondrial function in OA based on endogenous molecular targets has been accomplished. In addition, stem cells and exosomes have been deeply researched in the context of cartilage regeneration, and these factors both reverse mitochondrial dysfunctions. Thus, we hypothesize that biomedical approaches will be applied to the treatment of OA. Furthermore, we have summarized the global status of mitochondria and osteoarthritis research in the past two decades, which will contribute to the research field and the development of novel treatment strategies for OA.

## Introduction

Osteoarthritis (OA), a chronic and progressive cartilage degeneration disease (1) with a high morbidity and disability rate (2), is characterized by cartilage degeneration, osteophyte formation, thickening of subchondral bone, synovial inflammation, and meniscal injuries (3). As the global population ages and the proportion of obese people increases, the morbidity of OA continues to rise. At present, ~250 million people suffering from OA worldwide bear a tremendous economic burden as does society (4). OA tends to occur in the elderly population; cellular senescence is a contributor to age-related diseases (5), and studies have shown that OA is typical representatives of age-related diseases (6). Alleviating pain is the main purpose of non-surgical treatment, but this treatment does not alleviate the progression of OA (7).

Chondrocytes are the only cell type present in mature cartilage and change pathologically when OA occurs (8). Multiple factors can lead to OA, including inflammatory cytokines, mechanical stress, ageing, metabolic factors, and other pathological changes, which could increase reactive oxygen species (ROS) (9), induce oxidative stress in mitochondria, cause mitochondrial DNA (mtDNA) damage, result in mitochondrial damage, and shorten the life span of chondrocytes (10). The loss of mitochondrial membrane potential (MMP) leads to a reduction in energy production, an increase in the permeability of the mitochondrial membrane (11), and the release of apoptotic factors such as cytochrome C (Cyt-C), apoptosis-inducing factor, and procaspases from the mitochondria into the cytoplasm. Obvious changes in the morphology and function of mitochondria have been shown in ageing cells, and mitochondrial dysfunction is a key factor in cellular senescence (5, 12), demonstrating that mitochondria may be a therapeutic target for anti-ageing treatment and reduce the morbidity of OA in the elderly population (13). In addition, mitochondrial genetics are indispensable in the pathogenesis of OA. The accumulation of somatic mutations in mtDNA is a major contributor to human ageing and degenerative diseases (14). Reducing mtDNA damage, including the integrity of mtDNA4977, could optimize mitochondrial function, and maintain the homeostasis of chondrocytes. Furthermore, the mitochondrial apoptotic pathway has been implicated in chondrocyte apoptosis in OA (15). More specific therapeutic strategies on the basis of an in-depth molecular understanding of OA are thus essential (16).

With the research and application of stem cells and exosomes in cartilage repair, biomedical approaches to optimize mitochondrial function will be the preferred method for the thorough treatment of OA. Furthermore, gene therapy is also booming, and we therefore think that biological measures to modify the disease will be the major approach for OA treatment. In the present article, we have reviewed mitochondrial dysfunction mainly in the context of OA chondrocytes and summarized the endogenous molecular targets related to mitochondrial function. Moreover, research progress on exogenous drugs for the treatment of OA by restoring mitochondrial function in chondrocytes has been reviewed. In addition, we have described the global status of mitochondrial and OA research, which may contribute to predicting the trend in mitochondrial research regarding the treatment of OA. Furthermore, these findings will be instructive for mechanistic research on mitochondrial functions in OA, contributing to fundamental research on the treatment of OA through the mitochondrial pathway and providing novel strategies for the clinical treatment of OA.

## Biological Function of Mitochondria

**Figure 1:**
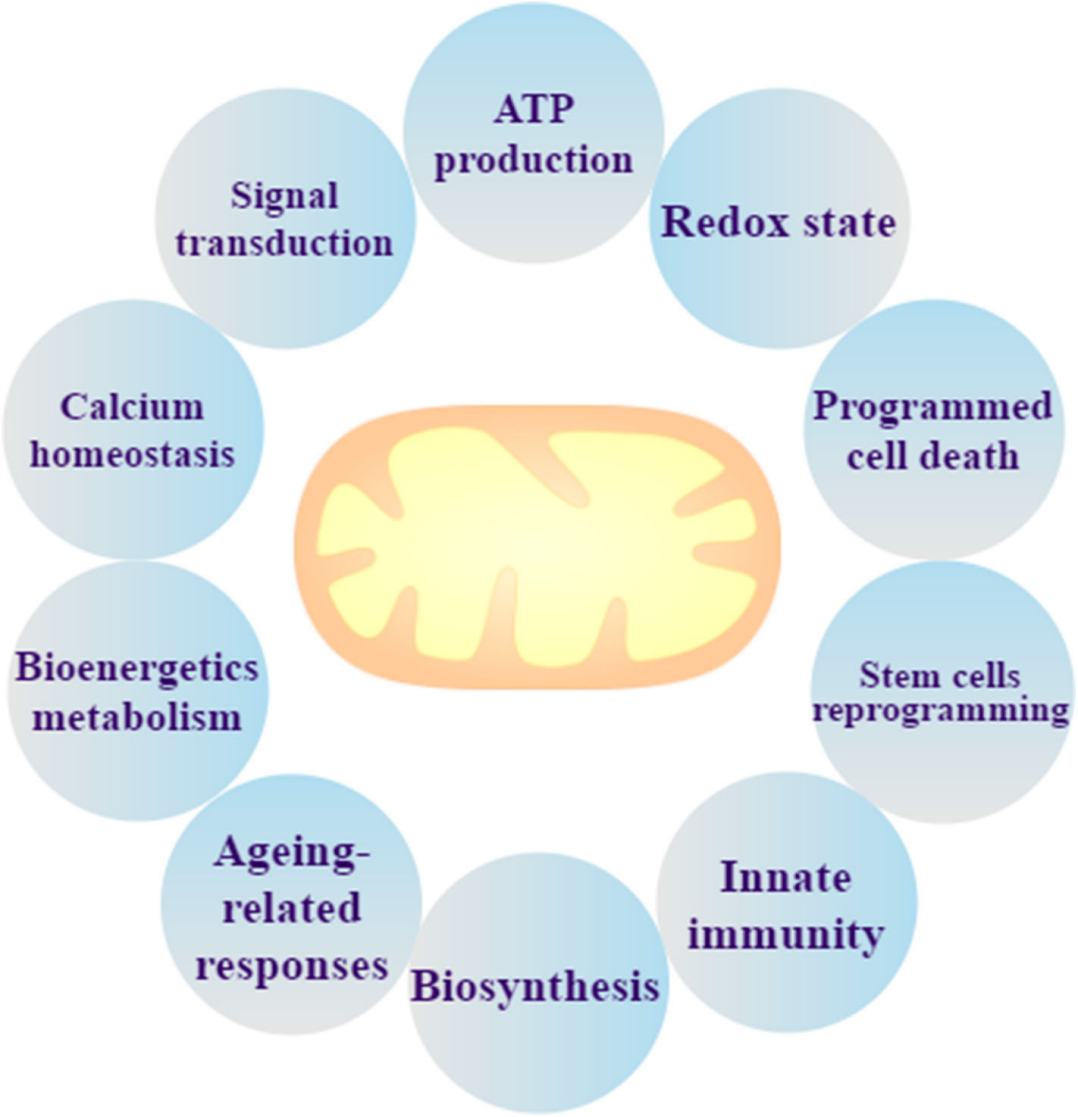
Mitochondrial biological function. Mitochondria are not only the organelle for ATP production and signal transduction, but they can also maintain the redox state and calcium homeostasis, regulate programmed cell death, and perform bioenergetics metabolism, stem cells reprogramming, ageing-related responses, innate immunity, and biosynthesis.

Mitochondria, encapsulated by bilayer membranes, are remarkably dynamic organelles and considered as the “powerhouse” of eukaryote cells. Mitochondria not only generate the energy required for cellular metabolism by oxidative phosphorylation (OXPHOS), but they also produce heat in certain specialized cell types, such as brown adipocytes (17). Approximately 2,000 mitochondria within a eukaryotic cell occupy ~20% of the cell volume (12). There are protein complexes in the inner mitochondrial membrane that transfer and pump protons through the mitochondrial respiratory chain (MRC) for ATP production, such as NADH dehydrogenase (complex I), succinate dehydrogenase (complex II), Cyt-C reductase (complex III), and Cyt-C oxidase (complex IV). Pyruvate and fatty acids could be converted to acetyl CoA by mitochondria, and CoA is metabolized by the citric acid cycle to produce NADH (18) where energy electrons are used to produce ATP (19). In addition to ATP production, intermediate metabolites for biosynthesis, protein modifications, signal transduction, programmed cell death, bioenergetic metabolism, the redox state, calcium homeostasis, innate immunity, stem cell reprogramming, and ageing-related responses (20–22) occur in the mitochondria (17) (Figure 1).

**Figure 2:**
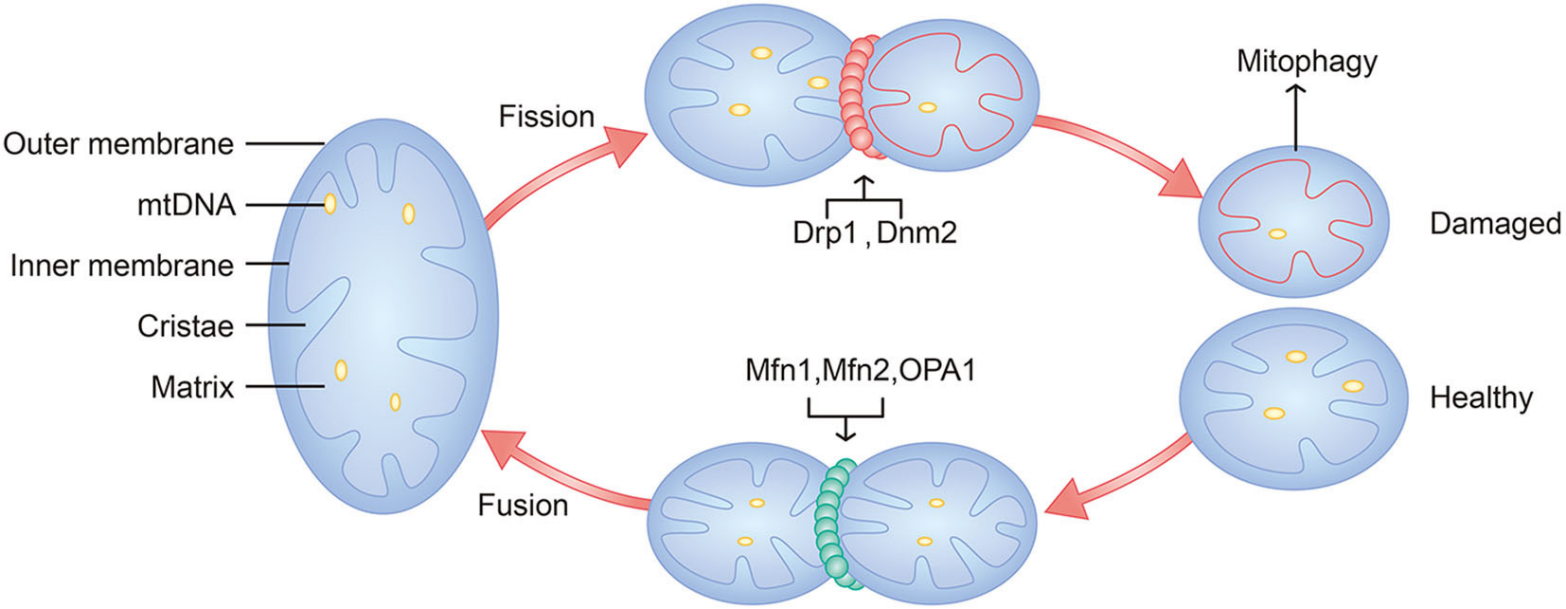
Schematic diagram of mitochondria and mitochondrial dynamics. Major components of mitochondria include outer membrane, inner membrane, cristae, matrix, and mtDNA. Mitochondrial fusion is mediated by Mfn1, Mfn2, and OPA1. Mitochondrial fission is mediated by Drp1, Dnm2. Damaged mitochondria will undergo mitophagy.

Recently, more research has focused on mitochondrial dynamics. The dynamic characteristics consist of mitochondrial fusion, mitochondrial fission and mitophagy (36), which are crucial for normal mitochondrial function and are critically associated with mitochondrial biogenesis and mitophagy (37). Mitofusins 1 (Mfn1) and Mitofusins 2 (Mfn2) mediate the fusion of the outer membrane, and optic atrophy 1 (OPA1) mediates the fusion of the inner membrane (38). Dynamin-related protein 1 (Drp1) and classical dynamin 2 (Dnm2) are the main mediators of mitochondrial fission (39) (Figure 2). When mitochondrial fission becomes increasingly dominant, damaged mitochondria undergo mitophagy in chondrocytes in the context of OA (40, 41), which could cause mitochondria to fail to produce sufficient bioenergy, regulate calcium and maintain the redox state. In contrast, mitochondrial fusion could enhance the biological function of mitochondria, which could make chondrocytes energetic and inhibit apoptosis.

In normal chondrocytes, mitochondria play a role in regulating signaling by modulating the redox state, supplying cofactors for biochemical reactions, such as molecular chaperones to facilitate protein folding, and generating ligands for signal transduction, such as AMPK signaling and calcium signaling (17, 42, 43). Calcium stored in mitochondria is helpful for maintaining calcium homeostasis in cells, and mitochondria are dedicated to transport extracellular matrix (ECM) calcium (12, 44). The mineralization of cartilage has been confirmed to involve calcium phosphate-containing granules, which are known as “matrix vesicles” (45). Moreover, Professor Alexandra E. Porter and colleagues found that mitochondrial granules contribute to the transport of clusters of calcium and phosphate ions to the ECM to facilitate mineralization, and Professor Lehninger AL suggested that mitochondria could release calcium phosphate to the ECM to take part in bone formation (46, 47). In addition, Professor Brian Glancy and colleagues showed that calcium activated nearly every step within the electron transport chain (ETC) (48) and activated enzymes, such as NADH, Cyt-C, complex III, and complex IV, in the pathways of oxidative metabolism in mitochondria (49, 50). Furthermore, mitochondria could regulate and balance the apoptosis by initiating cell death (17).

## Mitochondrial Dysfunction in Osteoarthritis

**Figure 3:**
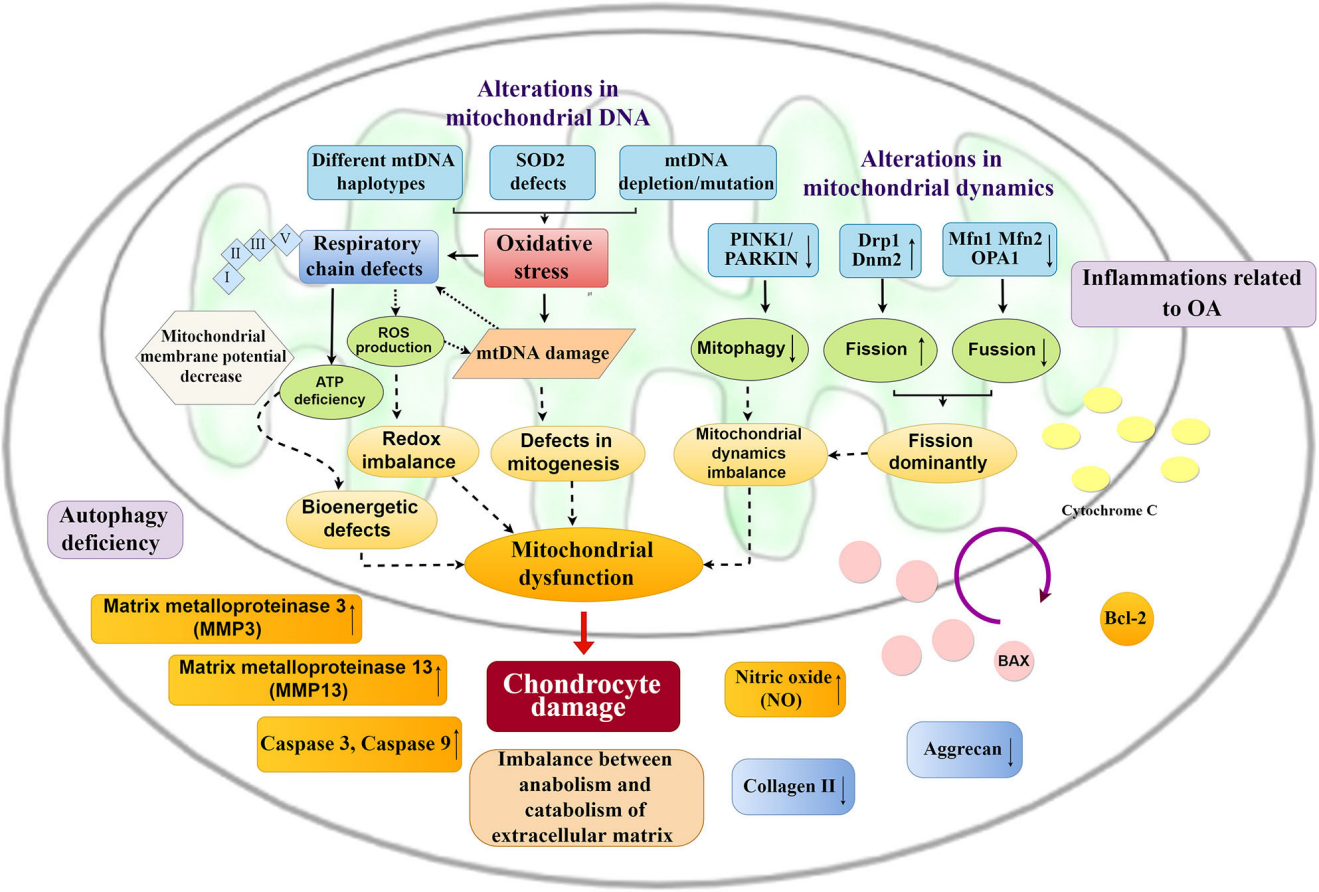
Mitochondrial changes and chondrocyte damage in OA. Alternations in mitochondrial DNA and mitochondrial dynamics cause a series of mitochondrial changes. Excessive oxidative stress, respiratory chain defects and imbalance of mitochondrial dynamics result in mitochondrial dysfunction, which could promote chondrocyte damage, including inflammatory injury, imbalance between anabolism and catabolism of the extracellular matrix, and an increase in apoptosis of chondrocytes.

Mitochondrial dysfunction mainly manifests as decreased ATP production, increased oxidative stress, calcium dysregulation, increased permeability of the mitochondrial membrane, and mtDNA alternations, which result in cartilage degeneration. Chondrocyte damage occurs and is mainly reflected in the increases in MMP-3, MMP-13, NO, and inflammatory injury with an imbalance between catabolism and anabolism of extracellular matrix (51), including reductions in aggrecan and collagen II, which eventually induce OA (Figure 3).

### Decreased ATP Production

Mitochondrial dysfunction can lead to a decrease in the activity of respiratory chain complexes I, II, III, and V, the loss of MMP, and decreases in OXPHOS in OA chondrocytes (52), which could induce chondrocytes to release interleukin-1β (IL-1β) and lead to inflammation (12). Two primary mechanisms of ATP production include substrate phosphorylation in the glycolytic pathway and in the tricarboxylic acid (TCA) cycle and OXPHOS occur at the inner membrane (53). ATP production is driven by the transmembrane proton gradient. An inflammatory response in chondrocytes with the upregulation of cyclooxygenase 2 (COX-2) and prostaglandin E2 (PGE2) production could be generated by ETC dysfunction (54). Both TNF-α and IL-1β inhibit the activity of ETC complex I (55), which induces decreases in ATP production and MMP. Inhibiting the ETC thus could decrease ATP synthesis (56).

### Increased Oxidative Stress

Mitochondrial dysfunction maintains a positive regeneration circle with oxidative stress, increased ROS, and mtDNA damage, which are regarded as hallmarks of chronic degenerative diseases (57). The accumulation of ROS and mtDNA damage can activate the nuclear factor-κB (NF-κB) pathway, which is the main regulator of inflammation (57). Avascular and hypoxic tissue are always used to describe cartilage, and chondrocytes are the only cell types in articular cartilage that maintain the balance of extracellular matrix (ECM) synthesis and degradation (58). ROS, as by-products of oxidation-reduction reactions, are generated in the MRC (59). A lower level of ROS is beneficial for maintaining chondrocyte homeostasis, and a higher level of ROS induces the depolarization of mitochondrial membrane, which could lead to sustained ROS production (60). An initial theory suggested that ROS have deleterious effects on ageing and degenerative diseases (61). Accumulating evidence has demonstrated that increased oxidative stress and the overproduction of ROS, including superoxide anion, hydrogen peroxide (H_2_O_2_), and nitric oxide (NO), play pivotal roles in the pathogenesis of OA (10, 62). The overproduction and accumulation of ROS and ATP deficiency decrease mitogenesis and break the redox balance. DNA and especially mtDNA could be injured (63). Oxidative stress could damage the mitochondrial respiratory chain protein complexes in chondrocytes (12). Due to the accumulation of ROS in chondrocytes, the decrease in collagen and glycosaminoglycan synthesis and the enhancement of metalloproteinases and aggrecanases induce chondrocytes to undergo a switch from anabolic to a catabolic gene expression, which results in cartilage breakdown (34). Furthermore, the depletion of superoxide dismutase 2 (SOD2), the major mitochondrial antioxidant protein, occurs in early cartilage degradation and could exacerbate inflammation and enhance ROS, contributing to OA progression (64, 65). Mitochondria are the dominant intracellular organelles in charge of the generation of ROS (66). ROS overload induced by oxidative stress results in the loss of MMP by stimulating the mitochondrial permeability transition pore (PTP) (67). High levels of cholesterol are naturally present in the cell membrane of chondrocytes, and chondrocytes could produce their own cholesterol and synthesize all the indispensable proteins for cholesterol biosynthesis (68, 69). Hypercholesterolemia animal models with changes in cartilage have been studied by Mao et al. (69), and the researchers demonstrated the direct effect of high cholesterol on cartilage degeneration and chondrocyte hypertrophy. When exposed to the synovial fluid with raised cholesterol levels, chondrocytes could be damaged because of the changes in the fluidity of the cell membrane and activation of membrane lipid signaling pathways (70). There is a close relationship between increased cholesterol oxidation products and mitochondria-derived oxidative stress, which leads to increased production of mitochondrial ROS (69), and Mao et al. showed that the cholesterol-lowering drug and the mitochondria-specific antioxidant have protective effects on attenuating OA symptoms caused by high cholesterol, such as atorvastatin and Mito-TEMPO.

### Calcium Dysregulation

Calcium, a ubiquitous intracellular second messenger, is involved in numerous cellular processes (71, 72). Calcium overload can lead to ROS overproduction, mitochondrial depolarization, MMP damage, and apoptosis (73). The maintenance of intracellular calcium homeostasis is achieved by mitochondrial uptake of calcium through a uniport transporter and the release of calcium through the inositol-1,4,5-trisphosphate receptor (IP_3_R), the sodium/calcium exchanger, or through the PTP, which is stimulated by excessive calcium in the mitochondrial matrix (72, 74). The PTP is a large conductance channel in the inner membrane of mitochondria (75), and both high levels of calcium and ROS can activate the PTP opening (76). The PTP makes the membrane non-specifically permeable to any molecule up to 1.5 kDa, including protons, and the mitochondria cannot maintain a pH gradient or MMP any longer (77, 78). The PTP leads to the collapse of MMP, leading to mitochondrial swelling and release of calcium and Cyt-C, ultimately stimulating apoptosis (8, 72). Calpains are calcium-activated proteases that could destroy the sodium/calcium exchanger and result in calcium overload and cell death (79). Furthermore, calcium overload and the activation of BAX by calpains lead to mitochondrial depolarization (72, 80).

### Increased Permeability of the Mitochondrial Membrane

Chondrocyte apoptosis induced by inflammation, oxidative stress, and increased mitochondrial membrane permeability (81) is positively associated with the degree of cartilage damage (82, 83). The collapse of the MMP leads to mitochondrial depolarization (8), which causes mitochondrial swelling, outer mitochondrial membrane collapse, and release of Cyt-C (84, 85). The BAX/mitochondrial Cyt-C/Caspase signaling pathway is shown to be associated with chondrocyte apoptosis (31). The downregulation of Bcl-2, the increase in expression of BAX, Caspase-3, and Caspase-9, and the increase in permeability of mitochondrial membrane can promote the outflow of Cyt-C from mitochondria into the cytoplasm and the inflow of BAX from the cytoplasm into mitochondria, increasing chondrocyte apoptosis. When damaged by various oxidative stimuli, the initiation of chondrocyte apoptosis induced by increased ROS is promoted (10, 15, 86). Studies have shown that mitochondrial dysfunction with reduced MMP and increased mitochondrial membrane permeability could promote the migration of Cyt-C from the mitochondrial matrix to the cytoplasm (87), which could induce apoptosis due to the activation of caspases and increase the BAX/Bcl-2 ratio (88). Moreover, the level of ROS in mitochondria is significantly increased (89), which could induce oxidative stress, destroy cartilage homeostasis, and increase chondrocyte apoptosis (60). The balance of mitochondrial dynamics could inhibit the apoptosis induced by oxidative stress (90, 91).

### mtDNA Mutation

In addition to mitochondrial dysfunction, the inheritance of mitochondria also acts as a pivotal role in the process of OA (92). mtDNA, a 16,569 bp circular and double-stranded molecule, encodes 13 protein subunits for the respiratory chain and 24 RNA components (22 tRNAs and 2 rRNAs) for mitochondrial protein synthesis (93). Chondrocytes from OA patients exhibit higher levels of mtDNA damage than chondrocytes from normal individuals (94). mtDNA damage could be caused by the increased ROS burden of aged chondrocytes (63, 95). At the same time, the accumulation of mtDNA mutations above a critical level could lead to dysfunction of the respiratory chain and increased ROS production, which could promote excessive chondrocyte apoptosis and enhance inflammatory responses (8). mtDNA haplogroups modulate crucial functions such as ATP production, oxygen consumption, ROS generation, and the expression of mitochondrial and nuclear genes (96). mtDNA haplotype J is associated with a lower risk of knee osteoarthritis (KOA) compared to that of mitochondrial mtDNA haplotype H (97). The mtDNA haplotype may be a biomarker for OA diagnosis and prognosis, and be closely involved in the OA phenotype (98). Therefore, the pattern of latent drugs that mimic the physiological effects of mtDNA haplotype J may be a potential treatment strategy for OA (99).

## Endogenous Molecular Targets to Reverse Mitochondrial Dysfunction

**Figure 4:**
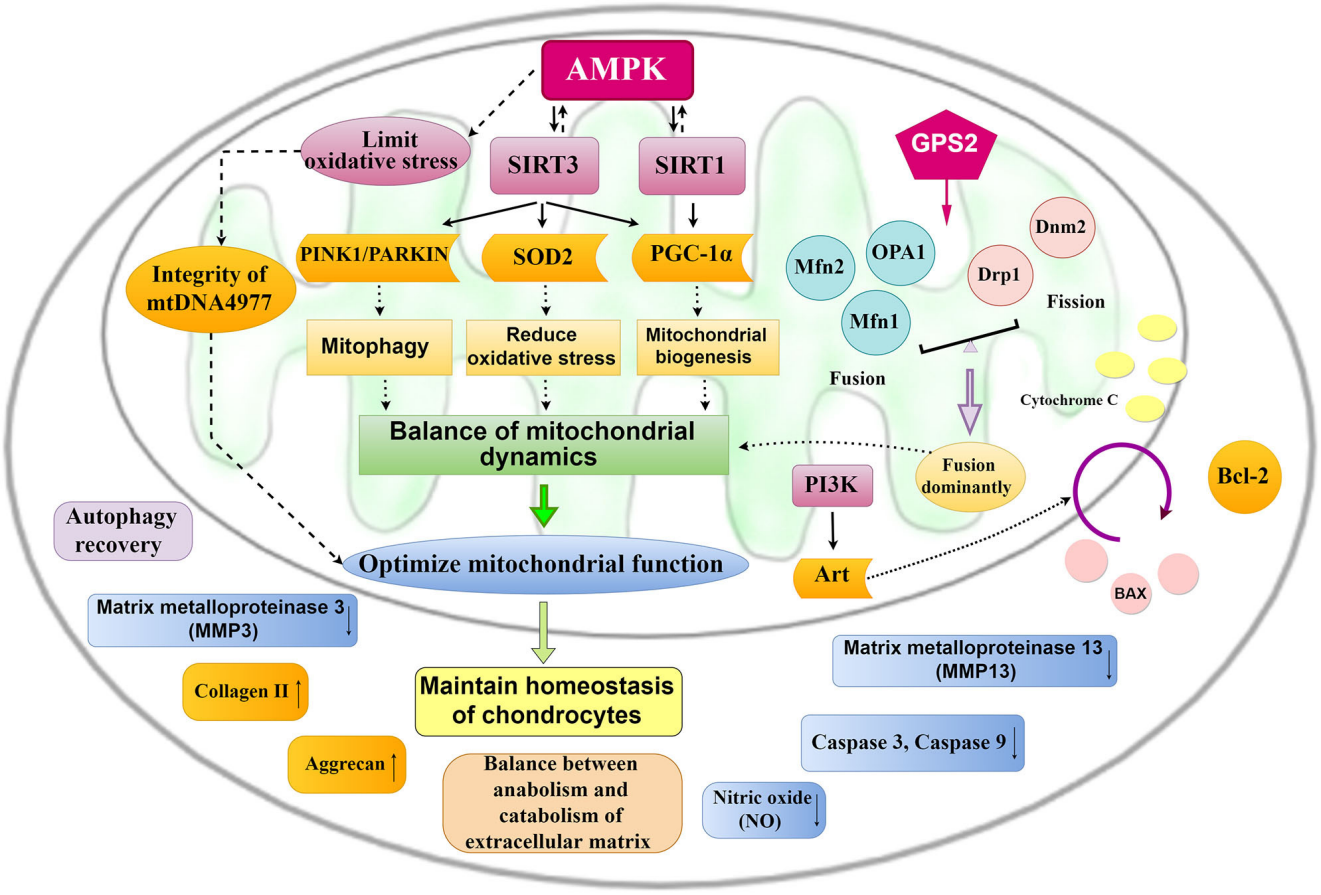
Mitochondrial pathways for the treatment of OA. The activation of AMPK-SIRT-3/ SIRT1-PGC-1α, AMPK-SIRT-3-SOD2, and AMPK-SIRT-3-PINK1/PARKIN signaling could promote mitochondrial biogenesis and mitochondrial fusion and reduce oxidative stress, which contribute to optimize mitochondrial function and then maintain chondrocyte homeostasis.

The vital role of mitochondrial changes in the development of OA has been demonstrated (8, 12, 52, 100), and endogenous molecular targets that optimize mitochondrial dynamics and morphology will turn into potential targets for OA treatment (Figure 4). AMPK, Sirtuin, PGC-1α, PINK1, PARKIN, and Nrf2 are endogenous molecules, and the activation of AMPK/SIRT1/3/PGC-1α, AMPK/SIRT3/SOD2, and AMPK/SIRT3/Parkin/PINK1 signaling could promote mitochondrial biogenesis and reduce oxidative stress, contributing to balancing mitochondrial dynamics and improving MMP. Moreover, upregulating OPA1, Mfn1, and Mfn2 and downregulating Drp1 and Dnm2 through GPS2 could promote mitochondrial fusion to enhance mitochondrial biological functions.

### AMPK

Adenosine 5′-monophosphate (AMP)-activated protein kinase (AMPK), the serine/threonine kinase, is a key regulator to adapt to changes in energy demand (101). When in a hypoxic state, AMPK can be activated and phosphorylate multiple downstream targets, promoting the inhibition of ATP-consuming pathways and the activation of the ATP-producing pathway (101, 102). Dysregulation of AMPK has been associated with a variety of age-related diseases related to mitochondrial dysfunction and imbalance of cellular energy, including diabetes, atherosclerosis, cardiovascular disease, cancer, neurodegenerative diseases, and OA (103, 104), suggesting the translational potential of pharmacological AMPK activators to limit OA progression (52, 102). In chondrocytes, activation of AMPK suppresses NF-κB activation, oxidative stress, and multiple inflammatory and catabolic responses (104). Moreover, AMPK could regulate both mitochondrial biogenesis and mitophagy to balance mitochondrial dynamics (52).

### Sirtuin

AMPK activity regulates energy metabolism via downstream mediators, including the nicotinamide adenine dinucleotide (NAD+)-dependent deacetylases Sirtuin1 and Sirtuin3 (SIRT1 and SIRT3, respectively). The key role of AMPK in the treatment of OA through the mitochondrial pathway and mitochondrial acetylation-induced OA has been identified, while SIRT3 is the main deacetylase in mitochondria, and SIRT3 activation can protect cells by regulating mitochondrial dynamics and mitophagy. SIRT1/3 and AMPK regulate each other (105). Increasing evidence shows that SIRT1 is significant in promoting mitochondrial dysfunction and OA progression (83). It has been proven that SIRT1 enzymatic activity is necessary for cartilage homeostasis (64, 65). The loss of SIRT1 in chondrocytes also leads to increases in MMP-13, apoptotic markers, and NF-κB, resulting in the accelerated OA development (83, 106). Upregulation of SIRT1 can inhibit the activation of COX-2, MMP-13, and NF-κB-induced TNF-α and decrease the upregulation of MMP-13 and acetylation of NF-κB p65 induced by IL-1β (83, 107). SIRT1 is a strong inducer of autophagy (108), which is reduced in OA, and therapeutic enhancement of autophagy is chondroprotective *in vitro* and *in vivo* (52, 109, 110). The NAD+-dependent deacetylase Sirtuin3 is the major deacetylase in mitochondria (111), contributing to the regulation of the mitochondrial antioxidant system and adenosine-triphosphate (ATP) production (112). Depletion of the mitochondrially localized antioxidant superoxide dismutase 2 (SOD2) promotes mitochondrial dysfunction and increased production of ROS (64, 65). A study showed that mitochondrial acetylation could promote the development of OA, while SIRT3 could enhance the antioxidant capacity of chondrocytes by enhancing the activity of SOD2 (113). Moreover, SIRT3 could activate and enhance the activity of AMPK in chondrocytes, which could reduce the loss of mtDNA4977 and maintain mtDNA integrity, thereby improving the function of mitochondria and protecting chondrocytes (28). Studies have shown that mitophagy can eliminate damaged mitochondria isolated by mitochondrial fission, which is a cytoprotective mechanism to maintain mitochondrial stability and quality (114). Moreover, the relationship between mitophagy and OA has been confirmed (60, 115). SIRT3 depletion can reduce mitophagy (116) and SIRT3 activation protects cells by regulating mitochondrial dynamics and mitophagy (117). Drugs that can activate SIRT3 may therefore be potential treatments for OA through the mitochondrial pathway.

### PGC-1 α

The mitochondrial biogenesis master regulator peroxisome proliferator–activated receptor γ coactivator 1α (PGC-1α) acts by inducing the transcription of nuclear respiratory factors (NRFs) (e.g., NRF-1 and NRF-2) (52), thereby increasing the expression of mitochondrial transcription factor A (TFAM) and other nuclear-encoded mitochondrial respiratory complex subunits (118, 119). TFAM is induced to translocate to mitochondria, which stimulates mitochondrial DNA replication and mitochondrial gene expression, thus stimulating the biogenesis of mitochondria (52, 118). It is well-known that SIRT1 and its substrate PGC-1α regulate aspects of energy metabolism through mitochondria (83). PGC-1α activity is regulated by phosphorylation and NAD 1-dependent deacetylation via metabolic biosensors AMPK, SIRT1, and SIRT3 (52, 120). Furthermore, Zhao et al. showed that PGC-1α is essential for mediating AMPK activity to block catabolic responses and suppress oxidative stress in chondrocytes (118).

### Parkin/PINK1

Autophagy is closely related to apoptosis in the pathogenesis of numerous degenerative diseases, and studies have shown that autophagy is inhibited in OA chondrocytes (121). Autophagy is a mechanism of intracellular catabolism through which cells can remove dysfunctional organelles and macromolecules to prevent the occurrence of cell stress, preventing mitochondrial dysfunction (122). Lotz et al. called the process mitophagy, which eliminates damaged mitochondria and prevents oxidative stress (123). Parkin, an E3 ubiquitin ligase and mitochondrial outer membrane (OMM) protein, operates in conjunction with PTEN-induced kinase 1 (PINK1), and phosphorylation of Parkin by PINK1 transforms it into an active phospho-ubiquitin-dependent E3 ligase, which can respond to the loss of MMP (ΔΨM) to eliminate damaged mitochondria (124). The evidence that Parkin-mediated clearance of damaged mitochondria limits the generation of ROS and prevents the induction of oxidative stress in OA chondrocytes was first demonstrated by Mohammad et al. (60).

### Nrf2

Nuclear transcription factor erythroid-2-like factor 2 (Nrf2) plays a chondroprotective role in OA and can suppress metalloproteinase expression induced by IL-1β (125). Nrf2 is a redox-sensitive transcription factor that positively regulates the expression of antioxidant and cytoprotective enzymes, including HO-1, NQO1, GST, SOD, GPx, and CAT (35, 126). Nrf2/antioxidant response element (ARE) signal transduction is one of the crucial antioxidant systems to maintain the redox state and has been regarded as a strategy to eliminate the damage caused by excessive ROS production (99, 127). Heme oxygenase-1 (HO-1), a ARE regulated by Nrf2, has been reported to prevent diseases caused by oxidative stress as a major therapeutic target of Nrf2 (128).

## Exogenous Drugs to Optimize Mitochondria in OA

The presence of the antioxidant defense system to avoid mitochondrial dysfunction and excessive chondrocyte apoptosis is extremely limited (129). Research on exogenous drugs to improve mitochondrial function in OA based on endogenous molecular targets is thus necessary (Table 1).

**Table 1:** Mitochondrial pathways for the treatment of OA.

**Potential drugs**	**Cells**	**Methods**	**Mechanism**	**Effects**	**References**
Melatonin	Chondrocytes (CHON-001)	*In vitro*: Co-culture *In vivo*: Histological evaluation	Inhibit PI3K/Akt, JNK, ERK, p38 and MAPK	Inos↓, COX-2↓, NO↓, PGE_2_↓	(23)
Resveratrol	Chondrocytes	*In vitro*: Co-culture	BAX/mitochondrial Cyt-C/Caspase	COX-2↓, NO↓, PGE_2_↓	(24)
DHM	TNF-α-treated chondrocyte and rats	*In vitro*: Co-culture *In vivo*: Histological evaluation	AMPK/SIRT3/PGC-1α	Mitochondrial fusion↑, antioxidant capacity ↑, ECM balance↑	(25)
Apple procyanidins	Primary chondrocytes and chondrocyte-specific Sod2^−/−^ mice	*In vitro*: Co-culture *In vivo*: Histological evaluation	AMPK/SIRT1/PGC-1α	Integrity of mtDNA↑, mitochondrial biogenesis↑ and proteoglycan biosynthesis↑	(26)
25 μM Zinc	MIA-treated SW1353 chondrocytes	*In vitro*: Co-culture	PINK1-Mitophagy PI3K/Akt/Nrf2	Mitophagy↑, oxidative stress ↓	(27)
SIRT3 activator	Human and mouse chondrocytes; C57BL/6 male mice	*In vitro*: Co-culture *In vivo*: Histological evaluation	AMPK/SIRT3/SOD2	Integrity of mtDNA4977↑	(28)
Quercetin	Chondrocytes from 1-week-old Sprague Dawley rats; OA rats.	*In vitro*: Co-culture *In vivo*: Histological evaluation	AMPK/SIRT1; Inhibit caspase-3	NO↓, MMP-3↓, MMP-13↓ and apoptosis↓	(29)
Puerarin	MIA-treated OA rats	*In vivo*: Histological evaluation	AMPK/PGC-1α	Mitochondrial biogenesis↑	(30)
LRWXG	ACLT-treated rats	*In vivo*: Histological evaluation	BAX/mitochondrial Cyt-C/Caspase	Bcl-2↑, MMP-3↓ and MMP-13↓	(31)
Ginsenoside Rg1	IL-1β-treated chondrocytes	*In vitro*: Co-culture	PI3K/Art	Caspase-3↓, TIMP-1↑, MMP-13↓ and Bcl-2↑	(32)
CS	H_2_O_2_-treated chondrocytes	*In vitro*: Co-culture	Increase MMP	MMP↑, Caspase-3↓ and Caspase-9↓	(33)
200 μM taurine	H_2_O_2_-induced chondrocytes	*In vitro*: Co-culture	Regulate Nrf2, miR-146a and miR-34a	Bcl-2↑, BAX↓	(34)
DADS	C2812 chondrocytes	*In vitro*: Co-culture	Enhance Nrf2	GPx1↑, GPx3↑, GPx4↑, CAT↑, SOD1↑, BAX/Bcl-2↓ and Caspase-3↓	(35)

### Antioxidants

Appropriate antioxidant strategies and the discovery of antioxidants are essential to protect chondrocytes against oxidative stress (86, 130, 131). Recent studies have shown that melatonin, dihydromyricetin, quercetin, taurine, and diallyl disulfide all act as antioxidants and are potential drugs for the treatment of OA.

#### Melatonin

Melatonin (N-acetyl-5-methoxytryptamine), an amine hormone produced by the pineal gland of mammals, is a broad-spectrum antioxidant and free radical scavenger (132). Melatonin and its metabolites can remove ROS by radical scavenging and improve the activation of antioxidant enzymes, thus regulating inflammation, proliferation, apoptosis and metastasis (133). Various experiments have demonstrated that melatonin can inhibit the phosphorylation of PI3K/Akt and MAPKs (23) and inhibit the loss of MMP and the release of mitochondrial Cyt-C (134). Kim et al. (23) demonstrated that melatonin acts as a potent inhibitor of H_2_O_2_-induced inducible nitric oxide synthase (iNOS) and cyclooxygenase-2 (COX-2) gene expression while also suppressing the production of NO and PGE_2_ in human chondrocytes, and the researchers thought that the inhibitory effect of melatonin on cartilage degeneration may be associated with the SIRT1 pathway.

#### Dihydromyricetin

Dihydromyricetin (DHM), which is mainly composed of flavonoids, can scavenge free radicals and has anti-inflammatory and antioxidative effects (135). SIRT3 can be activated by DHM through the AMPK/SIRT3/PGC-1α signaling pathway and can enhance mitochondrial fusion, maintain mitochondrial function and the homeostasis of chondrocytes, improve the antioxidant capacity of chondrocytes, and increase aggrecan and collagen II levels (25). DHM can also promote mitophagy to protect chondrocytes by activating SIRT3, which provides a new treatment strategy for OA.

#### Quercetin

Quercetin, a flavonoid compound, is widely found in vegetables and fruits and possesses antioxidant properties. Studies have revealed that quercetin is a potent anti-atherosclerotic drug as a result of its anti-inflammatory and antioxidative capacities (136). A study showed that quercetin could be used for the treatment of OA rats and demonstrated that quercetin could reverse mitochondrial dysfunction, improving MMP, oxygen consumption, and ATP production. The induction of glutathione (GSH) and glutathione peroxidase (GPX) by quercetin eliminated excessive ROS, which reduced or even abolished oxidative stress (29). Moreover, quercetin inhibited the accumulation of nitric oxide (NO), matrix metalloproteinase 3 (MMP-3), and MMP-13 produced by inflammation through AMPK/SIRT1 signaling, playing a key role in the inhibition of extracellular matrix degeneration. Quercetin also decreased chondrocyte apoptosis by inhibiting the caspase-3 signaling pathway (7). Therefore, quercetin is a potential therapeutic drug for OA that acts through the mitochondrial pathway.

#### Taurine

Taurine (2-aminoethane sulfonic acid), another antioxidant that is highly effective in attenuating free radical toxicity, has been identified (137). Taurine can ameliorate ROS-induced chondrocyte damage and exert chondroprotective properties, including the deposition of extracellular matrix components and proliferation of chondrocyte (138). Sara et al. (34) showed that 200 μM taurine could reduce mitochondrial superoxide anion production by activating Nrf2 and promote an increase in anti-apoptotic Bcl-2 and a reduction in proapoptotic BAX to inhibit chondrocyte apoptosis (139). In addition, the regulation of miR-146a and miR-34a expression in OA chondrocytes was first demonstrated. Taurine may be a potential drug for OA.

#### Diallyl Disulfide

Diallyl disulfide (DADS), a main component of garlic with antioxidant and anti-inflammatory properties (35, 140), could reduce pro-inflammatory cytokines expression, such as TNF-α, IL-1β, inducible nitric oxide synthase (iNOS), and COX-2 (35), by inhibiting the nuclear factor-κB (NF-κB) signaling pathway (141). Moreover, the pivotal etiological role of apoptosis in cartilage degeneration and the antioxidant and anti-apoptotic properties of DADS were considered (126, 140), the mechanism of DADS in oxidative stress and consequent apoptosis induced by IL-1β in C2812 human chondrocytes was studied by Hosseinzadeh et al. (35). The findings demonstrated that DADS protected C2812 chondrocytes against oxidative stress and reduced ROS and NO production by enhancing Nrf2 nuclear translocation. In addition, DADS markedly enhanced the expression of GPx1, GPx3, GPx4, CAT, and SOD1 and decreased the ratio of BAX/Bcl-2 and Caspase-3 activation to inhibit apoptosis (35). DADS could therefore be extracted and developed a potential drug for OA, and an interesting perspective emerged that a diet rich in garlic might be beneficial to reduce both the incidence and progression of OA.

### Inhibiting the Mitochondrial Apoptotic Pathway

#### Resveratrol

The natural polyphenolic compound resveratrol (polystilbene, C_14_H_12_O_3_), a non-flavonoid polyphenol compound with anti-inflammatory and antioxidative properties, is mainly derived from grape leaves, grape skin, and various fruits (142). Mitochondrial dysfunction increased the inflammatory response to cytokines in human chondrocytes and resveratrol significantly reduced the inflammatory response (143). Resveratrol alleviated the chondrocyte damage induced by interleukin-1β (IL-1β) through the NF-κB signaling pathway (144). Moreover, resveratrol has been regarded as a potent activator of SIRT1, which can prevent human chondrocyte apoptosis under cellular stresses, including nutritional stress, catabolic stress, and mechanical shear stress, by promoting Bcl-2 translocation to mitochondria and inhibiting BAX translocation to mitochondria (145). The optimization of mitochondrial function in animal models and protection against IL-1β-induced chondrocyte apoptosis can be achieved by resveratrol (24).

#### Xanthan Gum

Xanthan gum (XG), an extracellular acidic polysaccharide, is released by the fermentation of Xanthomonas (146, 147). Studies have shown that the BCL2-associated X protein (BAX)/Cyt-C/Caspase signaling pathway contributes to cartilage degeneration (88). A low range of molecular weights of XG (LRWXG) has been applied for rabbit OA treatment (31). In this study, the inhibition of cartilage matrix destruction and the protection of subchondral bone were demonstrated. In addition, LRWXG could inhibit the formation of small pores in the mitochondrial inner membrane and inhibit the swelling and rupture of the mitochondrial outer membrane, which could stabilize membrane potential and the permeability of the mitochondrial membrane. Moreover, activation of Bcl-2 and inhibition of BAX activity were achieved by LRWXG. Both of these factors could reduce the translocation of Cyt-C from mitochondria to the cytoplasm (31). The decrease in Cyt-C in the cytoplasm downregulated Caspase-3 and Caspase-9 in chondrocytes, which reduced the formation of apoptotic bodies and decreased chondrocyte apoptosis. Xintian Shao, the author of the study, therefore thought that LRWXG could inhibit chondrocyte apoptosis by conditioning the BAX/mitochondrial Cyt-C/Caspase signaling pathway and protect chondrocytes from degeneration.

#### Chondroitin Sulfate

Chondroitin sulfate (CS), a glycosaminoglycan that is widely extracted from animal and fish cartilage, is an essential component of the extracellular matrix (148). A study indicated that carp chondroitin sulfate increased MMP and inhibited the levels of Caspase-3 and Caspase-9 by reducing mitochondrial fission, which decreased chondrocyte apoptosis (33). It appears that chondroitin sulfate also has the potential to treat OA through the mitochondrial pathway.

#### Ginsenoside Rg1

Ginsenoside Rg1 (Rg1) is one of the most active components in ginseng along with steroidal saponin (149). The therapeutic effect of Rg1 on nervous system diseases and cardiovascular diseases has been reported, which inspired Huang et al. to investigate whether Rg1 protected chondrocytes (32). Their findings showed that Rg1 could enhance Bcl-2 expression, advance tissue inhibitor of metalloproteinase-1 (TIMP-1) expression, inhibit Bax activity, inhibit MMP-13 synthesis, and inhibit Cyt C release from mitochondria to the cytosol through enhancing phosphatidylinositol 3-kinase (PI3K)/Akt signaling, which inhibited Caspase-3. The inhibition of Caspase-3 led to the inhibition of chondrocyte apoptosis and protected chondrocytes. Rg1 may thus be a potential treatment for OA treatment through the PI3K/Akt/mitochondrial signaling pathway.

### Enhanced Mitochondrial Dynamics

#### Apple Polyphenols

Apple polyphenols from immature apples, compounds composed of several polyphenols, exert anti-allergy, anti-fatigue and life-extending effects (26, 150). Masuda et al. investigated the role of apple polyphenols in protecting chondrocytes and improving OA (26). Their findings showed that apple polyphenols could enhance mitochondrial biogenesis by promoting the integrity of mtDNA and mitochondrial fusion through AMPK/SIRT1/PGC-1α signaling. Moreover, apple polyphenols could promote proteoglycan biosynthesis. In an *in vivo* study, apple procyanidins protected against articular cartilage degeneration and prevented the development of knee OA in chondrocyte-specific Sod2-/- mice (26). Based on these results, we can conclude that apple polyphenols may be potential drugs for treating OA.

#### Puerarin

Puerarin, an isoflavone derivative, is isolated from the Chinese medicine Pueraria and possesses antioxidative, anti-inflammatory, anticancer and vasodilating effects (151). The ability of puerarin to restore mitochondrial dysfunction has been confirmed (152). Furthermore, puerarin could reduce mitochondrial dysfunction and damage to chondrocytes by increasing mitochondrial biogenesis and restoring mitochondrial function through the upregulation of AMPK/PGC-1α signaling, which protected chondrocytes in OA (30).

#### Zinc (25 μM)

In a study of metformin for the treatment of OA, Chenzhong Wang found that metformin could improve the expression of SIRT3 in chondrocytes and activate the PINK1 (PTEN induced putative kinase 1)/Parkin signaling pathway and could ameliorate mitochondrial function and protect chondrocytes from OA by promoting mitochondrial fusion and eliminating dysfunctional mitochondria through mitophagy (89). Huang et al. showed that 25 μM zinc could protect chondrocytes injured by monosodium iodoacetate (MIA) through the PINK1-dependent selective mitophagy pathway, which indicated that 25 μM zinc was protective against OA (27).

## Global Status of Mitochondrial and Osteoarthritis Research

We collected 361 papers, and the dataset from Jan. 2000 to Dec. 2019 was derived from the Web of Science (WOS) Core Collection, which is regarded as the optimum database (153). The search terms were as follows: [(TS = (mitochondria^*^ AND osteoarthritis)] OR [TS = (mitochondrion^*^ AND osteoarthritis))] AND (Language = English) AND (Document type = Article AND Review). The logistic growth model f(*x*) = a/[1 + e^b−cx^], where x is the year and f(x) represents the cumulative quantity of papers by year, was used to model the cumulative volume of documentation because of its great fitness and ability to predict future trends (154). VOS viewer (Leiden University, Leiden, Netherlands) were tools used to develop the co-occurrence analysis map (155).

### Global Status

**Figure 5:**
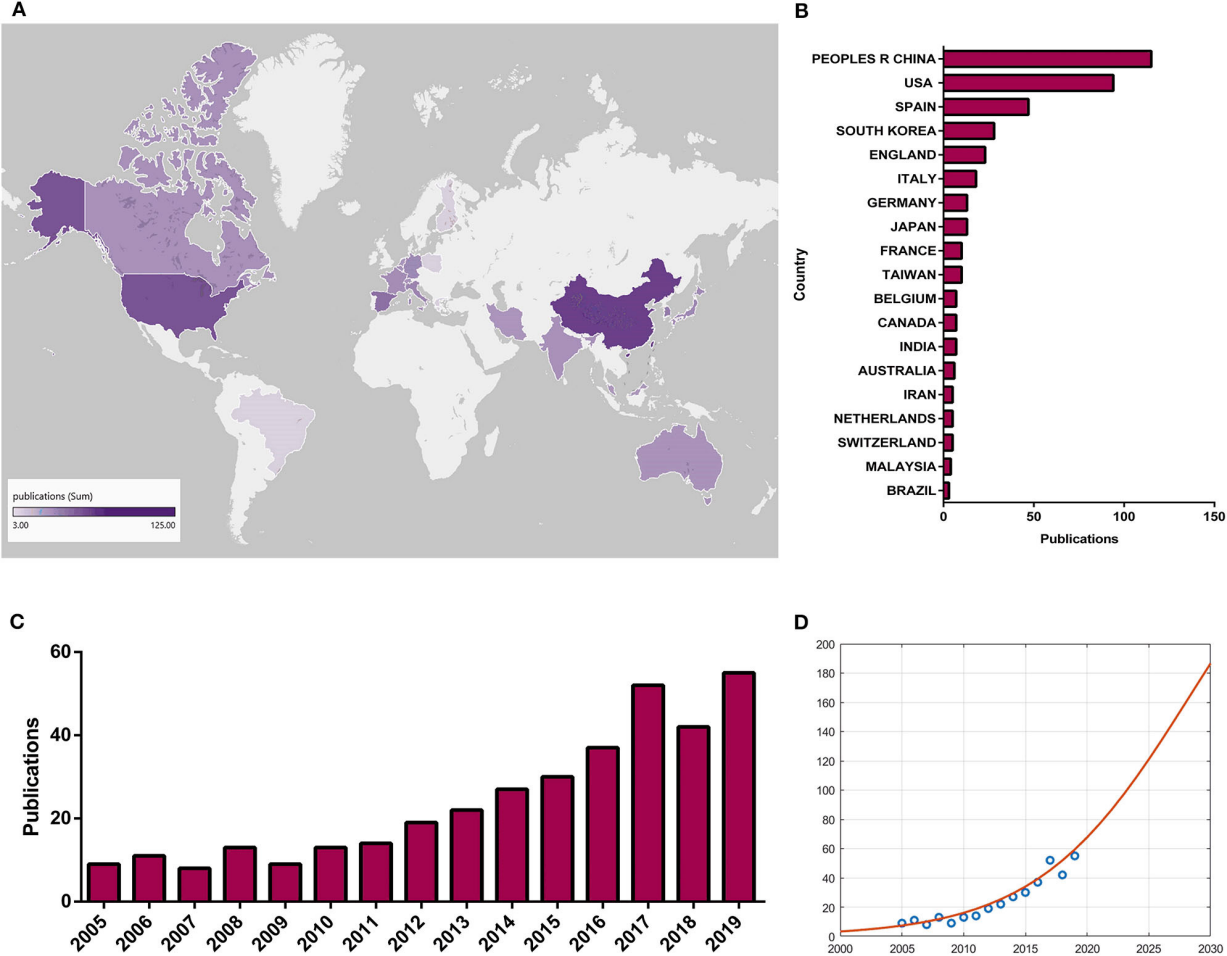
Global trends and contributed countries on mitochondria and OA research. **(A)** World map showing the distribution of mitochondria and OA research, in which the different color depths represent the different numbers of publications in different countries. **(B)** The sum of publications related to mitochondria and OA research from 20 countries or regions. **(C)** The annual number of publications related to mitochondria and OA research in the past 20 years. **(D)** Model fitting curves of growth trends of accumulated number of publications on mitochondria and OA research.

Variations in the quantity of academic publications in a certain research field are a significant indicator of the development trend (155). Determining the number of papers within a period of time and guiding multivariate statistical analysis are conducive to the research level and future trends (155). The global status of mitochondrial and osteoarthritis research has demonstrated that research on mitochondria and OA has been a popular topic in the field. An total of 361 papers from 2000 to 2019 were obtained from the WOS database on the basis of the search formula. Over the past 20 years, there has been a growing trend in global publications, which showed that the relative research on mitochondria and OA increased and the total number of publications significantly increased (Figure 5C). Moreover, Figure 5D shows the logistic regression model meeting curves f(*x*) = 330/[1 + e^328.2543−0.1618x^] of the quantity of papers on mitochondria and OA research in the future per year. The top 20 productive countries are listed in Figure 5B due to the total quantity of papers per country. China was the largest contributor with the highest number, and Figure 5A shows the top 25 countries that made the greatest contributions to mitochondrial and OA research globally. The darker the color, the greater the quantity of papers.

### Co-occurrence Analysis

**Figure 6:**
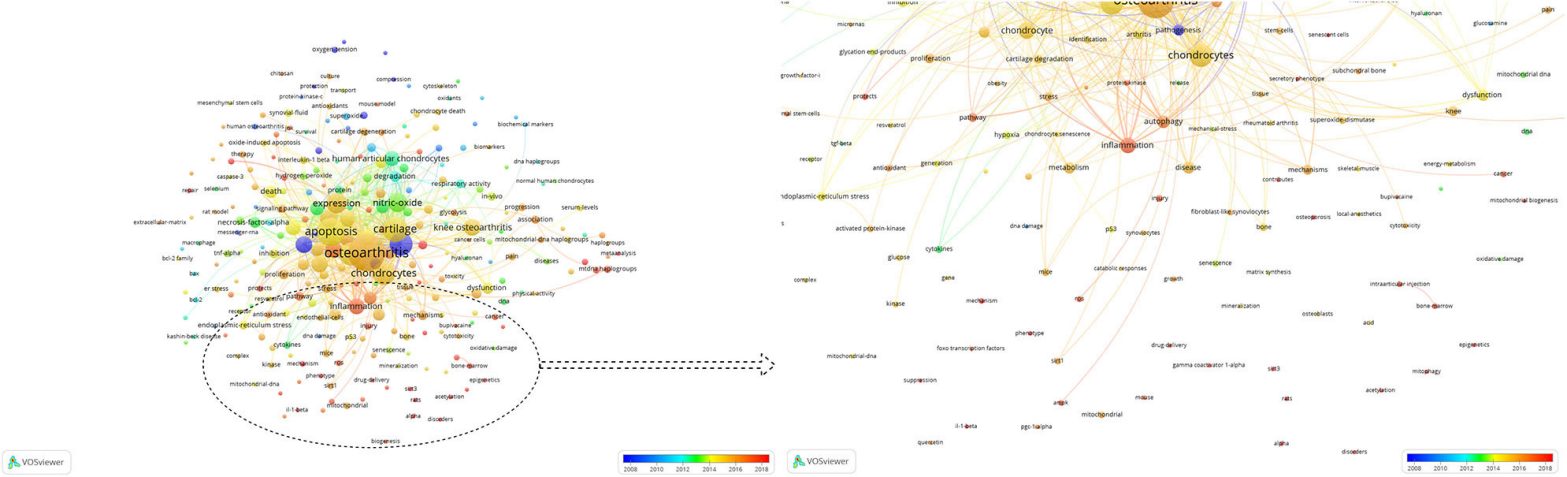
Co-occurrence analysis of global research about mitochondria and OA. Mapping of keywords in the research on mitochondria and OA. The size of the points represents the frequency. Distribution of keywords according to the mean frequency of appearance. Keywords in blue appeared earlier than those in yellow and red colored keywords appeared later.

The purpose of co-occurrence analysis is to determine the relevance of items according to the quantity of projects that appear together and describe the internal relationships and structure of an academic field, and reveal the research frontiers (156). The development of scientific research and programs could be monitored and followed closely as popular topics and directions were identified through co-occurrence analysis (155, 157). Keywords were analyzed by VOS viewer, and 277 identified keywords are shown in Figure 6. The larger the spheres, the greater the frequency. It was obvious that “Apoptosis,” “Chondrocytes,” “Oxidative,” “Nitric-oxide,” and “Autophagy” had the highest frequency and may be the main research themes in the past two decades. In addition, the blue color means that the keywords occurred early, and red colored keywords occurred later. We found that “Phenotype,” “SIRT3,” “PCG-1α,” “AMPK,” “FOXO transcription factors,” “Mitophagy,” “Acetylation,” “Nrf2,” and “Repair,” which were red colored, occurred recently, which may mean that research on mitochondria and osteoarthritis will focus on mechanistic studies and cartilage repair.

## Conclusion and Perspectives

Research on mitochondria and OA is currently a popular topic. Mechanistic research on the relationship between mitochondria and OA has been launched, and corresponding research on the treatment of OA has also made excellent progress. Although articular cartilage deterioration is the main pathological characteristic of OA, it is now widely accepted that the entire joint, including the synovium, is involved (158). The synovium contributes to the general physiological function of joints and the regulation of the joint microenvironment by secreting synovial fluid to supply nutrients and lubricate the cartilage (159). Fibroblast-like synoviocytes (FLSs) are highly sensitive to hypoxia and reoxygenation (H/R), and IGFBP-3 is overexpressed in cartilage and synovial fluid under H/R conditions (160). The induction of nerve growth factor-induced gene B (NGFI-B, Nur77) by IGFBP-3 has been confirmed (161). The mitochondrial membrane permeability could be enhanced by Nur77, which results in the translocation of Cyt-C from the mitochondrial matrix to the cytoplasm and initiates an intrinsic and classic apoptosis pathway: the caspase pathway (162). Therefore, improving synovitis through the mitochondrial pathway may be a potential strategy for OA treatment (163).

Strategies for OA treatment are tiered, and non-pharmacological methods, including education and self-management, exercise, weight loss if overweight or obese, and walking aids as indicated, are widely recommended and regarded as first-line treatments (3, 164). The most commonly recommended pharmacological methods in the guidelines include paracetamol and NSAIDs (3, 165). In the context of surgery, joint replacement surgery, knee osteotomy, knee joint distraction and arthroscopic knee surgery (3), and autologous chondrocyte transplantation are currently the most effective treatments (166). The current pharmacological methods used to OA treatment are largely palliative (3), thus modifying OA progression, including slowing, halting, and reversing progression, are critical.

Biotherapy and gene therapy are current research trends in disease treatment. Stem cells, including mesenchymal stem cells (BMSCs), umbilical cord stem cells, embryonic stem cells and induced pluripotent stem cells, are regarded as exceptional donor cells for mitochondrial transfer, and numerous studies have confirmed the significance of mitochondrial transfer in stem cell therapy (167), especially BMSCs (168). Moreover, transplantation of stem cells has recently become a research hotspot in treating tissue injury. Whether stem cell transplantation can optimize mitochondrial function in OA is therefore worth exploring. Exosomes, which are extracellular vesicles 30–150 nm in diameter, have similar functions as those of derived cells without apparent side effects in both healthy and diseased cells (169), and studies have shown that the therapeutic effects of mesenchymal stem cells (MSCs) can be replicated by their secreted exosomes (52, 170). MSC-derived exosomes possess the biochemical potential to restore homeostasis in bioenergetics, cell number and immunomodulation (52, 171, 172). Exosomes contain mitochondrial membrane components and mtDNA (173). Zheng et al. investigated the ability of primary chondrocyte-derived exosomes to abrogate mitochondrial dysfunction in degenerated chondrocytes (170). The results indicated that exosomes from chondrocytes could reduce the expression of inflammatory cytokines, restore mitochondrial dysfunction, and reduce macrophage polarization toward an M2 phenotype, resulting in the repair of injured chondrocytes. This finding is in accordance with the treatment of a mouse OA model with chondrocyte exosomes. Collectively, primary chondrocyte exosomes are potential disease-modifying therapeutic agents for OA. We therefore thought biomedical measures would be efficient for the treatment of OA based on optimizing mitochondrial function. CRISPR/Cas9 is the most convenient gene-editing tool so far, widely used in human embryonic stem cells (hESCs) and their derivatives for basic and clinical research (16, 174). Deng et al. showed that MSCs without DiGeorge syndrome critical region 8 (DGCR8) could alleviate human MSC senescence and mouse osteoarthritis (16, 175). More efficient and targeted gene-editing tools need to be developed, which contribute to precise genetic and epigenetic regulation, such as activation or inhibition of target genes *in vivo* (16). We thus predict that gene therapy will be a radical therapeutic strategy for OA treatment.

There are still numerous mechanisms that need to be further explored. Pain is the main symptom of OA patients and a major driver of clinical decisions (3, 176); therefore, whether the new strategy targeting the mitochondrial pathway for OA has an effect on pain relief is still unclear in the current study. Microvesicles are popular for research on the mechanism of OA treatment and whether microvesicles can promote mitochondrial fusion and biosynthesis to reduce chondrocyte apoptosis is not known. At the same time, how drugs that affect the mitochondrial pathway in OA work in mitochondria, which are subcellular organelles, is unclear. It is widely accepted that mitochondria and the nucleus are in two-way communication, and the way mitochondria conduct signal transduction with the nucleus after exposure to a drug effects to inhibit cell apoptosis and protect cells is worth studying. With the continuous investment in mitochondrial and OA research worldwide, a new strategy targeting the mitochondrial pathway in OA will have great breakthroughs and will make a great contribution to the treatment of OA. The day is coming when we will provide subcellular, cellular, and tissue-level mechanistic and clinical evidence for the treatment of OA to provide a more comprehensive and efficient treatment for OA patients.

## Author Contributions

XM, LW, and CX made the review article structure. CX and LW are responsible for reviewing. XM and PF finished writing. All authors contributed to the article and approved the submitted version.

## Conflict of Interest

The authors declare that the research was conducted in the absence of any commercial or financial relationships that could be construed as a potential conflict of interest.
